# Early childhood development and associated factors among children aged 24–59 months in Tanzania: evidence from the 2022 Tanzania Demographic and Health Survey

**DOI:** 10.3389/fpubh.2026.1892860

**Published:** 2026-07-30

**Authors:** Erneus Ernest, Christopher Hariri Mbotwa

**Affiliations:** 1 Department of Paediatrics and Child Health, Mbeya College of Health and Allied Sciences, University of Dar es Salaam, Mbeya, Tanzania; 2 Department of Social Sciences, Mbeya College of Health and Allied Sciences, University of Dar es Salaam, Mbeya, Tanzania

**Keywords:** child development, developmental milestones, Early Childhood Development Index, ECDI2030, preschool children development, Tanzania

## Abstract

**Background:**

Early childhood development is a key foundation for later health, learning, and productivity. However, evidence using the Early Childhood Development Index 2030 (ECDI2030) remains limited in Tanzania. This study assessed developmental status and associated factors among children aged 24–59 months in Tanzania.

**Methods:**

This was a cross-sectional analysis of nationally representative data from the 2022 Tanzania Demographic and Health Survey (2022 TDHS). The study included children aged 24–59 months living with their biological mothers during the study period. Developmental status was assessed using the UNICEF ECDI2030, with children classified as developmentally on track using age-specific recommended cut-off scores (24–29 months ≥7; 30–35 months ≥9; 36–41 months ≥11; 42–47 months ≥13; 48–59 months ≥15). Generalized linear models with Poisson distribution, log link, and robust variance estimation were used to assess factors associated with early childhood development. Adjusted prevalence ratios (aPRs) with 95% confidence intervals (CIs) were reported.

**Results:**

A total of 4,930 children with a mean age (±standard deviation) of 39.9 months (±10.0) were included for analysis. Overall, 47.0% of children were developmentally on track. The proportion declined with increasing age, from 57.6% among children aged 24–35 months to 35.8% among those aged 48–59 months. Female children had higher prevalence of being developmentally on track than males (aPR 1.15, 95% CI 1.07–1.23). Higher prevalence was observed among children of biological mothers aged 25–34 years (aPR 1.11, 95% CI 1.01–1.22) and 35–49 years (aPR 1.12, 95% CI 1.01–1.24), those whose biological mothers had primary (aPR 1.33, 95% CI 1.19–1.50) or secondary and higher education (aPR 1.43, 95% CI 1.25–1.65), children from rich households (aPR 1.18, 95% CI 1.06–1.31), and those whose biological mothers owned phones (non-smartphone: aPR 1.14, 95% CI 1.04–1.24); smartphones: aPR 1.27, 95% CI 1.12–1.44). Geographical disparities were observed, with higher prevalence in the Northern, Central, and Lake zones and Zanzibar, whereas lower prevalence was observed in the Western, Southern, and South West Highlands zones when compared with the Eastern zone.

**Conclusion:**

Nearly half of children aged 24–59 months were developmentally on track. Interventions addressing socioeconomic inequalities, caregiver education and geographical disparities are needed to improve early childhood development in Tanzania.

## Introduction

Early childhood development (ECD) refers to the process of development across the cognitive, physical, language, motor, social, and emotional domains from conception to 8 years of age (1). The period from conception through the first 3 years of life is particularly important for brain development because the brain develops rapidly, with neural connections forming at a rapid rate during this period (2). These neural connections are shaped by children’s experiences and their environment, laying the foundation for lifelong learning, health, and wellbeing (2, 3). Consequently, exposures during early childhood can have lasting effects across the life course, influencing educational achievement, health, productivity, and future earning potential (3, 4).

Investing in early childhood development benefits not only individual children but also societies by promoting inclusive growth, reducing inequities, and helping break intergenerational cycles of poverty (5). Despite growing global recognition of these benefits and substantial progress in child health and survival, the burden of poor developmental outcomes remains high, with an estimated 250 million children under 5 years in low- and middle-income countries (LMICs) at risk of not reaching their developmental potential (4).

In response, the World Health Organization (WHO), United Nations Children’s Fund (UNICEF), World Bank, and other partners have prioritized ECD through initiatives such as the Nurturing Care Framework. This framework serves as a roadmap to support parents, caregivers, and governments in providing children with the conditions needed to survive, thrive, and reach their full potential (5).

To translate these commitments into measurable progress, reliable and standardized indicators are needed to monitor child development outcomes and identify disparities (3). Although several assessment tools exist, they differ in the developmental domains assessed, methods of administration, target age groups, and suitability for generating nationally representative estimates (6). The earlier Multiple Indicator Cluster Surveys Early Childhood Development Index (MICS ECDI) was an important milestone, but it did not fully align with Sustainable Development Goal (SDG) indicator 4.2.1 and mainly targeted children aged 3–4 years (3). To address these gaps, UNICEF developed the Early Childhood Development Index 2030 (ECDI2030). It is the official measure for SDG indicator 4.2.1 for children aged 24–59 months, which estimates the proportion of children who are developmentally on track and provides internationally comparable data to guide policies and programmes. The ECDI2030 was specifically developed and validated for children aged 24–59 months. Consequently, developmental status in children younger than 24 months is not assessed using this standardized instrument because methodological work to develop a population-level measure for children aged 0–23 months had not yet been finalized when ECDI2030 was developed (3).

Marked disparities in developmental status persist across world regions. According to UNICEF global databases, the proportion of children aged 24–59 months who are developmentally on track is substantially higher in Eastern Europe and Central Asia (79%) than in Sub-Saharan Africa (54%). Even lower estimates were reported in Western and Central Africa (52%). Among countries classified as least developed by the United Nations, only 57% of children are developmentally on track (7). These findings indicate that many children in low-resource settings continue to face substantial barriers to achieving their developmental potential.

In Tanzania, a recent facility-based study conducted in four health facilities in Temeke Municipal, Dar es Salaam, reported that 34.1% of children aged 24–59 months were developmentally on track, but its findings may not represent the national population or geographic variation across the country (8). Consequently, nationally representative evidence on early childhood development status and associated factors in Tanzania remains limited. Therefore, this study aimed to determine the proportion of Tanzanian children aged 24–59 months who were developmentally on track and identify associated factors using ECDI2030.

## Materials and methods

### Study design

This was a cross-sectional analysis of data from the 2022 Tanzania Demographic and Health Survey (2022 TDHS). The 2022 TDHS is a nationally representative household survey that collects information on caregiver and child health, reproductive health, fertility, nutrition, malaria, and other population health indicators. Detailed methodology and survey procedures are available in the 2022 TDHS final report (9). This study was reported in accordance with the Strengthening the Reporting of Observational Studies in Epidemiology (STROBE) guidelines (10).

### Study population

The study included children aged 24–59 months. Children who had died before the survey and those not living with their biological mothers at the time of the survey were excluded from the analysis. For a mother with more than two children aged 24–59 months, only the youngest child was included.

### Sample design

The 2022 TDHS employed a two-stage stratified cluster sampling design based on the 2022 Tanzania Population and Housing Census sampling frame. In the first stage, 629 enumeration areas (EAs) (211 from urban areas and 418 from rural areas) were selected independently within urban and rural strata across all regions of mainland Tanzania and Zanzibar using probability proportional to size sampling. In the second stage, 26 households were systematically selected from each sampled EA, producing 16, 312 households. All women aged 15–49 years who were either permanent residents or visitors present in the selected households the night before the survey were eligible for interview. A total of 15,254 eligible women were interviewed. Women with children aged 24–59 months were eligible for ECDI2030 interview. The final sample comprised 4,930 children aged 24–59 months from mothers selected for ECDI2030.

### Study variables

#### Outcome variable

The outcome variable was early childhood developmental status measured using the ECDI2030. The ECDI2030 is a caregiver-reported population-based tool specifically developed and validated to assess whether children aged 24–59 months are developmentally on track according to age-specific developmental milestones and is therefore not applicable to children younger than 24 months (3). The index was constructed using 20 developmental items from the ECDI2030 module implemented in the standard TDHS questionnaire. A composite ECDI2030 score was generated by summing the developmental items, yielding a total score ranging from 0 to 20. Children were classified as developmentally “on-track” or “not on-track” based on the UNICEF-recommended age-specific cut-off points (24–29 months: ≥7; 30–35 months≥9; 36–41 months≥11; 42–47 months≥13; 48–59 months≥15) (3). Children meeting or exceeding the age-specific threshold were categorized as developmentally on-track.

#### Independent variables

The explanatory variables included sex of the child, biological mother characteristics (age, marital status, and education), household and community-level variables (geographical zone, place of residence, household wealth index, and telephone ownership). The household wealth index in the TDHS was constructed using principal component analysis of household assets and amenities and originally categorized into five wealth quintiles (poorest, poorer, middle, richer, and richest). For this study, the poorest and poorer quintiles were combined into a single “poor” category, while the richer and richest quintiles were merged into a “rich” category, resulting in three wealth index categories (poor, middle, and rich).

### Statistical analyses

Data were extracted from the children’s recode dataset (TZKR81FL). All analyses were done using Stata version 19 (StataCorp LLC, College Station, TX, USA). The complex survey design by weighting the data and taking into considerations clustering and stratification variables. Categorical variables were presented as frequencies and weighted percentages, while continuous variables were summarized using means and standard deviations. Associations between explanatory variables and developmental status were assessed using generalized linear models (GLM) with Poisson family distribution, log link function, and robust variance estimation. Both crude prevalence ratios (PRs) and adjusted prevalence ratios (aPRs) with their corresponding 95% confidence intervals were reported. Statistical significance was considered at *p* < 0.05.

### Ethics statement

Formal ethical approval for this secondary analysis was not required because the dataset is publicly available. Permission to use the data was obtained from the DHS Program. The 2022 TDHS received ethical approval from relevant national and international review boards, including the National Institute of Medical Research, the Zanzibar Medical Research Ethical Committee, the Institutional Review Board of ICF, and the Centers for Disease Control and Prevention, Atlanta. Informed consent was obtained from all participants before data collection.

## Results

### Socio-demographic characteristics

A total of 4,930 children aged 24–59 months with a male–female ratio of 1:1 were included in the analysis, with a mean age of 39.9 months (±10.0). The majority of biological mothers were in a marital union (4,152; 84.2%) and had attained primary education (2,652; 53.8%). Nearly three-quarters of the children resided in rural areas (3,573; 72.5%) (Table 1).

**Table 1:** Socio-demographic characteristics of study participants.

Variable	*n* (%)
Sex of child	
Male	2,486 (50.4)
Female	2,444 (49.6)
Age of a child (months)	
24–35	1,875 (38.0)
36–47	1,735 (35.2)
48–59	1,320 (26.8)
Biological mother’s age (years)	
15–24	1,048 (21.3)
25–34	2,332 (47.3)
35–49	1,550 (31.4)
Biological mother’s marital status	
In marital union	4,152 (84.2)
Previously in union	557 (11.3)
Never in union	221 (4.5)
Biological mother’s education level	
No formal education	1,052 (21.3)
Primary	2,652 (53.8)
Secondary+	1,226 (24.9)
Zone	
Eastern	476 (9.7)
Western	427 (8.7)
Northern	459 (9.3)
Central	460 (9.3)
Southern Highlands	352 (7.1)
Southern	166 (3.4)
South West Highlands	673 (13.7)
Lake	1,182 (24.0)
Zanzibar	735 (14.9)
Place of residence	
Urban	1,357 (27.5)
Rural	3,573 (72.5)
Household wealth index	
Poor	2,002 (40.6)
Middle	1,007 (20.4)
Rich	1,921 (39.0)
Telephone ownership	
No telephone	1,928 (39.1)
Non-smartphone	2,350 (47.7)
Smartphone	652 (13.2)

### Developmental status of children according to ECDI2030

**Figure 1:**
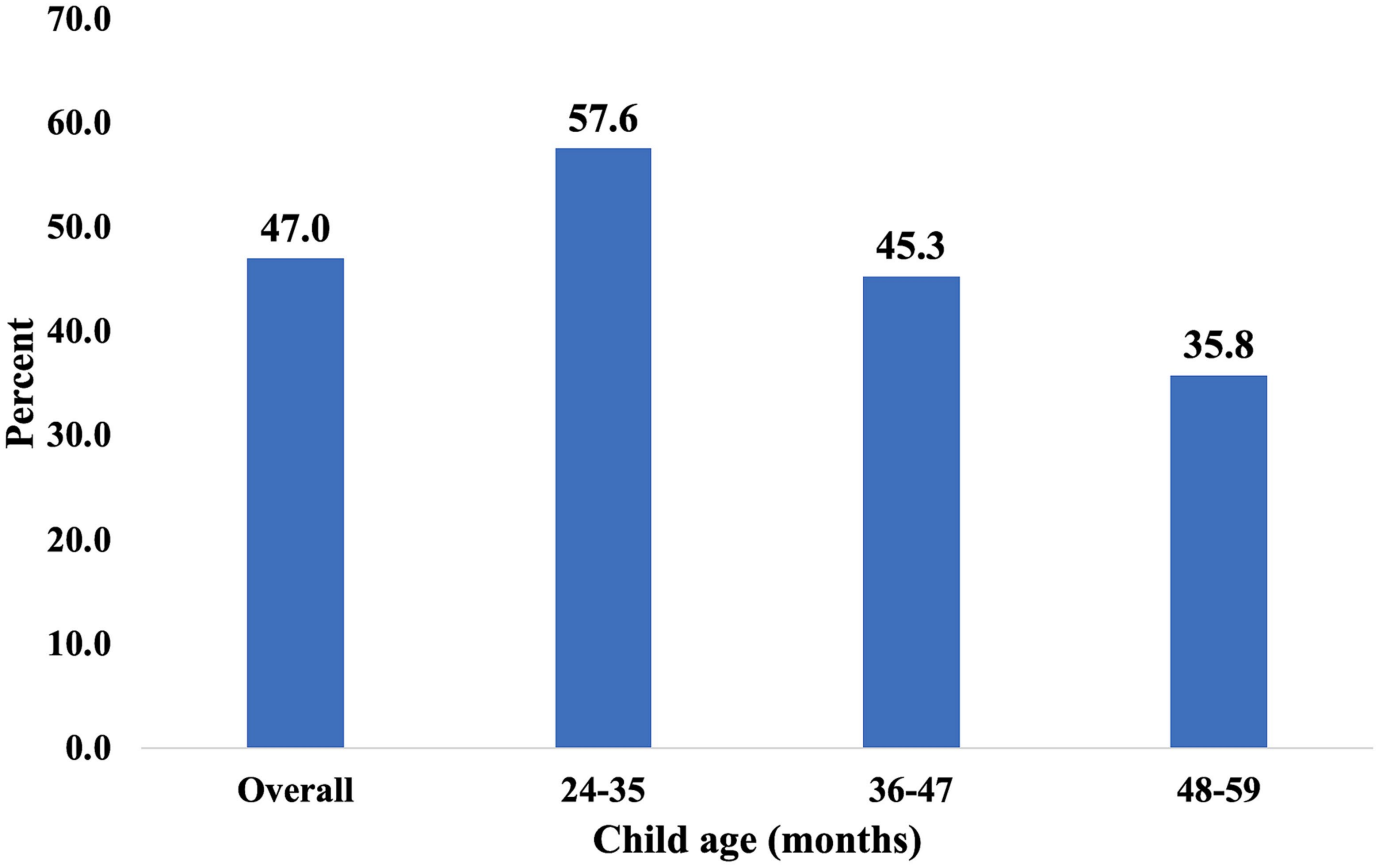
Percentage of children aged 24–59 months who were developmentally on track according to ECDI2030 by age group in Tanzania, 2022 TDHS.

Figure 1 presents the percentage distribution of children who were on track for ECDI2030. Overall, 47% of children were developmentally on track. The proportion of children on track decreased with increasing age, from 57.6% among those aged 24–35 months to 45.3% among those aged 36–47 months, and further to 35.8% among children aged 48–59 months.

### Factors associated with early childhood development

Table 2 presents the results of the bivariate and multivariable regression analyses. In the bivariate analysis, sex of the child, biological mother’s age, biological mother’s education level, geographical zone, place of residence, household wealth index, and telephone ownership were significantly associated with children being developmentally on track.

**Table 2:** Bivariate and multivariable analysis results.

Variable	*N*	% on track (weighted)	Bivariate analyses		Multivariable analysis	
			PR (95% CI)	*p*-value	aPR (95% CI)	*p*-value
Sex of child						
Male	2,486	44.1	Ref.	—	Ref.	—
Female	2,444	50.5	1.15 (1.06–1.23)	<0.001	1.15 (1.07–1.23)	<0.001
Biological mother’s age (years)						
15–24	1,048	41.0	Ref.	—	Ref.	—
25–34	2,332	50.3	1.23 (1.11–1.36)	<0.001	1.11 (1.01–1.22)	0.035
35–49	1,727	47.0	1.15 (1.03–1.28)	0.012	1.12 (1.01–1.24)	0.037
Biological mother’s marital status						
In marital union	4,152	48.0	Ref.	—	Ref.	—
Previously in union	557	42.4	0.88 (0.78–1.00)	0.048	0.91 (0.81–1.02)	0.117
Never in union	221	46.4	0.97 (0.81–1.15)	0.696	0.90 (0.76–1.07)	0.228
Biological mother’s education level						
No formal education	1,052	32.3	Ref.	—	Ref.	—
Primary	2,652	48.0	1.48 (1.32–1.66)	<0.001	1.33 (1.19–1.50)	<0.001
Secondary+	1,226	61.0	1.89 (1.67–2.13)	<0.001	1.43 (1.25–1.65)	<0.001
Zone						
Eastern	476	48.8	Ref.	—	Ref.	—
Western	459	20.8	0.43 (0.34–0.53)	<0.001	0.52 (0.42–0.65)	<0.001
Northern	459	59.6	1.22 (1.07–1.39)	0.003	1.33 (1.16–1.51)	<0.001
Central	460	55.5	1.14 (0.99–1.30)	0.064	1.27 (1.11–1.45)	0.001
Southern Highlands	352	48.4	0.99 (0.85–1.15)	0.913	1.02 (0.88–1.19)	0.756
Southern	166	25.9	0.53 (0.39–0.71)	<0.001	0.63 (0.47–0.84)	0.002
South West Highlands	673	30.5	0.62 (0.53–0.73)	<0.001	0.73 (0.62–0.86)	<0.001
Lake	1,182	53.2	1.09 (0.97–1.22)	0.160	1.20 (1.07–1.36)	0.003
Zanzibar	735	61.0	125 (1.10–1.42)	<0.001	1.15 (1.01–1.31)	0.038
Place of residence						
Urban	1,357	56.5	1.29 (1.19–1.39)	<0.001	0.99 (0.91–1.10)	0.929
Rural	3,573	43.8	Ref.	—	Ref.	—
Household wealth index						
Poor	2,002	38.3	Ref.			
Middle	1,007	43.9	1.14 (1.03–1.27)	0.011	1.03 (0.93–1.15)	0.538
Rich	1,921	58.7	1.53 (1.42–1.66)	<0.001	1.18 (1.06–1.31)	0.002
Telephone ownership						
No telephone	1,928	37.9	Ref.	—	Ref.	—
Non-smartphone	2,350	50.5	1.33 (1.22–1.45)	<0.001	1.14 (1.04–1.24)	0.005
Smartphone	652	66.4	1.75 (1.58–1.93)	<0.001	1.27 (1.12–1.44)	<0.001

In the multivariable analysis, sex of child, biological mother’s age, biological mother’s level of education, wealth index, ownership of telephone, and geographical zone were independently associated with being developmentally on track. Female children had a higher prevalence of being on track compared to males (aPR 1.15, 95% CI 1.07–1.23, *p* < 0.001). Children whose biological mothers were aged 25–34 years (aPR 1.11, 95% CI 1.01–1.22, *p* = 0.035) and 35–49 years (aPR 1.12, 95% CI 1.01–1.24, *p* = 0.037) had a higher prevalence of being on track compared with those from biological mothers aged 15–24 years. Children whose biological mothers had primary education (aPR 1.33, 95% CI 1.19–1.50, *p* < 0.001) and secondary or higher education (aPR 1.43, 95% CI 1.25–1.65, *p* < 0.001) had a higher prevalence of being on development track compared to those whose biological mothers had no formal education. There were significant variation across geographical zones, with higher prevalence in the Northern (aPR 1.33, 95% CI 1.16–1.51, *p* < 0.001), Central zones (aPR 1.27, 95% CI 1.11–1.45, *p* = 0.001), lake zone (aPR 1.20, 95% CI 1.07–1.36, *p* = 0.003), and Zanzibar (aPR 1.15, 95% CI 1.01–1.31, *p* = 0.038) and lower prevalence in the Western (aPR 0.52, 95% CI 0.42–0.65, *p* < 0.001), Southern (aPR 0.63, 95% CI 0.47–0.84, *p* = 0.002), and South West Highlands zones (aPR 0.73, 95% CI 0.62–0.86, *p* < 0.001), compared to the Eastern zone. Furthermore, children from rich households (aPR 1.18, 95% CI 1.06–1.31, *p* < 0.002) and those whose biological mothers owned non-smartphones (aPR 1.14, 95% CI 1.04–1.24, *p* = 0.005) or smartphones (aPR 1.27, 95% CI 1.12–1.44, *p* < 0.001) had a higher prevalence of being on track.

## Discussion

Nationally representative evidence on early childhood development status and associated factors in Tanzania remains limited. Using national representative data, this study assessed developmental status among children aged 24–59 months and identified associated factors.

We found that 47% of children were developmentally on track. The prevalence of being developmentally on track was higher among female children, those whose biological mothers were aged 25–34 years and 35–49 years, those whose biological mothers had higher education attainment, those from rich households, and those from households with telephone ownership. Differences were also observed across geographical zones. These findings highlight important disparities in early childhood development and may help inform targeted interventions and policies to improve developmental outcomes in Tanzania.

The proportion of children developmentally on track in this study was comparable to studies from some sub-Saharan African countries, including Benin (41%) and Eswatini (48%), but lower than those reported for Kenya (78%) and UNICEF’s sub-Saharan Africa estimate (54%) (7). These comparisons should be interpreted cautiously because the UNICEF database includes estimates derived from different survey years and measurement frameworks, including the earlier ECDI (mainly for children aged 36–59 months) and the newer ECDI2030 for children aged 24–59 months (7, 11). Overall, these findings highlight opportunities to strengthen nurturing care, early learning opportunities, and access to child development services in Tanzania.

Mother’s education was independently associated with the prevalence of children being developmentally on track in our study. Children were more likely to be developmentally on track with increasing biological mother’s education. This finding is consistent with studies conducted in Ghana and Bangladesh, where higher maternal education was also associated with better developmental outcomes (12, 13). Educated mothers may be more exposed to child development needs and practices, and better able to provide a favorable environment for child development at home and in school (12).

Similarly, household wealth was independently associated with children’s developmental status. Children from rich households were more likely to be developmentally on track than those from poor households. A Similar association between household wealth and developmental outcomes has also been reported in studies conducted in Ghana and Bangladesh (12, 13). In addition, a multicountry study showed that children with developmental delay were more likely to live in poverty than their peers in five of six surveyed countries (14). This association may reflect the influence of household wealth on families’ ability to provide key components of nurturing care, including adequate nutrition, access to health services, opportunities for early learning, and responsive caregiving (5).

Our findings indicate zonal disparities in the proportion of children who were developmentally on track, with higher prevalence in the Northern and Central zones, and lower prevalence in the Western, Southern, and South West Highlands, compared with the Eastern zone. Similar subnational variation in early childhood development has been reported in other LMIC settings, including Ghana and Bangladesh (12, 13). In Tanzania, the lower developmental status observed in the Southern and South West Highlands zones corresponds with reports of higher stunting burden in these same areas (15). Chronic undernutrition can adversely affect brain development, learning, and overall child functioning, and may partly explain the observed pattern. Other contributing factors may include differences in household socioeconomic conditions and access to supportive early childhood environments.

In our study, female children had a higher prevalence of being developmentally on track compared to male children. Similar findings were reported in two studies from Bangladesh (13, 16). Likewise, a multicountry analysis of 71 LMICs found that, where sex differences in early childhood development were observed, girls were more likely than boys to be developmentally on track, particularly in the social–emotional domain (17). This may partly reflect caregiver perceptions and reporting of child skills during caregiver interviews, gender-related expectations for girls’ and boys’ behavior, and differences in the stimulation and interactions girls and boys receive within the home environment (17).

Children born to biological mothers aged 25–34 years and 35–49 years had a higher prevalence of being developmentally on track compared with those born to biological mothers aged 15–24 years. Similar associations have been reported in a longitudinal study from the United Kingdom, where increasing maternal age was associated with better language development and fewer social and emotional difficulties in early childhood (18). In contrast, an Australian cohort study reported increased developmental vulnerability among children born to mothers aged above 35 years (19). Differences in study populations, developmental measures, and sociocultural contexts may partly explain these variations in findings. In our setting, older maternal age may reflect greater parenting experience, increased caregiving confidence, and more stable household environments, which could support early child development.

Telephone ownership among mothers was associated with a higher prevalence of children being developmentally on track. Evidence on the effects of telephone and other media use on early child development remains mixed. Interactive media, caregiver co-use of media with children, and educational content have been associated with enhanced language development (20). In contrast, excessive screen exposure has been associated with language delay, attentional difficulties, and poorer short-term memory outcomes (21). In our study, telephone ownership may reflect better household socioeconomic conditions and improved access to health, parenting, and child development information. However, these findings should be interpreted cautiously, as the study did not assess children’s direct use of digital devices, duration of screen exposure, or the quality of content accessed.

### Strength and limitations

This study has several strengths. It used nationally representative survey data, supporting the generalizability of the findings. The use of ECDI2030 provided a standardized measure of early childhood development. In addition, the large sample size, rigorous DHS survey methodology, and survey-weighted analyses strengthened the precision and reliability of the findings.

The study also has several limitations. First, the cross-sectional design of the 2022 TDHS limits causal interpretation of the observed associations. Second, the analysis was restricted to variables available in the 2022 TDHS dataset, and some potentially important biological, psychosocial, and environmental factors related to early childhood development were not assessed. Third, ECDI2030 domains were based on caregiver reports and may be affected by recall bias, reporting bias, or differences in the interpretation of child behaviors.

## Conclusion

Nearly half of children aged 24–59 months in this study were developmentally on track based on the ECDI2030. Being developmentally on track was associated with being a female child, biological mother’s age (25–34 years and 35–49 years), higher biological mother’s education attainment, living in a rich household, telephone ownership, and geographic variation, with higher prevalence in the Northern, Central, and Lake zones and Zanzibar, whereas lower prevalence was observed in the Western, Southern, and South West Highlands zones, compared with the Eastern zone. These findings may help guide ongoing efforts to strengthen nurturing care, early learning opportunities, and child development support services, with targeted support for population groups and areas with lower developmental outcomes.

## Data Availability

Publicly available datasets were analyzed in this study. This data can be found at: https://dhsprogram.com/Data/.
